# 5α-Reductase Type 1 Modulates Insulin Sensitivity in Men

**DOI:** 10.1210/jc.2014-1395

**Published:** 2014-05-13

**Authors:** Rita Upreti, Katherine A. Hughes, Dawn E. W. Livingstone, Calum D. Gray, Fiona C. Minns, David P. Macfarlane, Ian Marshall, Laurence H. Stewart, Brian R. Walker, Ruth Andrew

**Affiliations:** University/British Heart Foundation Centre for Cardiovascular Science (R.U., K.A.H., D.E.W.L., D.P.M., I.M., B.R.W., R.A.) and Clinical Research Imaging Centre (C.D.G.), University of Edinburgh, Queen's Medical Research Institute, Edinburgh EH16 4TJ, United Kingdom; and Radiology (F.C.M.) and Urology (L.H.S.) Departments, National Health Service Lothian University Hospitals Division, Western General Hospital, Edinburgh EH4 2XU, United Kingdom

## Abstract

**Context:**

5α-Reductase (5αR) types 1 and 2 catalyze the A-ring reduction of steroids, including androgens and glucocorticoids. 5α-R inhibitors lower dihydrotestosterone in benign prostatic hyperplasia; finasteride inhibits 5αR2, and dutasteride inhibits both 5αR2 and 5αR1. In rodents, loss of 5αR1 promotes fatty liver.

**Objective:**

Our objective was to test the hypothesis that inhibition of 5αR1 causes metabolic dysfunction in humans.

**Design, Setting, and Participants:**

This double-blind randomized controlled parallel group study at a clinical research facility included 46 men (20–85 years) studied before and after intervention.

**Intervention:**

Oral dutasteride (0.5 mg daily; n = 16), finasteride (5 mg daily; n = 16), or control (tamsulosin; 0.4 mg daily; n = 14) was administered for 3 months.

**Main Outcome Measure:**

Glucose disposal was measured during a stepwise hyperinsulinemic-euglycemic clamp. Data are mean (SEM).

**Results:**

Dutasteride and finasteride had similar effects on steroid profiles, with reduced urinary androgen and glucocorticoid metabolites and reduced circulating DHT but no change in plasma or salivary cortisol. Dutasteride, but not finasteride, reduced stimulation of glucose disposal by high-dose insulin (dutasteride by −5.7 [3.2] μmol/kg fat-free mass/min, versus finasteride +7.2 [3.0], and tamsulosin +7.0 [2.0]). Dutasteride also reduced suppression of nonesterified fatty acids by insulin and increased body fat (by 1.6% [0.6%]). Glucose production and glycerol turnover were unchanged. Consistent with metabolic effects of dutasteride being mediated in peripheral tissues, mRNA for 5αR1 but not 5αR2 was detected in human adipose tissue.

**Conclusion:**

Dual inhibition of 5αRs, but not inhibition of 5αR2 alone, modulates insulin sensitivity in human peripheral tissues rather than liver. This may have important implications for patients prescribed dutasteride for prostatic disease.

The 5α-reductases (5αRs) convert testosterone to its more potent metabolite 5α-dihydrotestosterone (DHT). Investigation of rare cases of 5αR deficiency, presenting with a 46XY disorder of sexual development, led to the discovery of 2 isozymes (1): 5αR type 1 (5αR1) is expressed in metabolic tissues including liver (2), adipose (3) and skeletal muscle (4), and 5αR type 2 (5αR2) is expressed predominantly in the reproductive tract, where deficiency accounts for disordered sexual development, and in human liver (2). 5αR inhibitors, which reduce circulating and prostatic DHT levels, are prescribed commonly in patients with benign prostatic hyperplasia (BPH). Finasteride inhibits 5αR2 selectively, whereas dutasteride inhibits both 5αR1 and 5αR2 (5, 6).

In addition to testosterone, 5αRs also catalyze reduction of a range of steroid hormones, including glucocorticoids (2). Due to widespread enzyme expression, and lack of substrate specificity, 5αR inhibition may alter local steroid concentrations in extraprostatic tissues. Targeting of another enzyme, 11β-hydroxysteroid dehydrogenase type 1, which metabolizes glucocorticoids in liver and adipose tissue, alters local but not systemic glucocorticoid levels and affects body fat distribution and insulin sensitivity (7, 8). Increased liver fat and decreased insulin sensitivity are seen in mice with targeted disruption of 5αR1, but not 5αR2 (9).

We hypothesized that inhibition of 5αR1 decreases insulin sensitivity in humans, as it does in rodents. Previous studies of the metabolic effects of 5αR inhibitors in humans have been limited to simple but insensitive measures such as fasting plasma glucose (10). To determine the influence of 5αR1, we compared the dual 5αR1 and 5αR2 inhibitor dutasteride with the 5αR2 selective inhibitor finasteride.

## Subjects and Methods

### Study design

**Figure 1.**
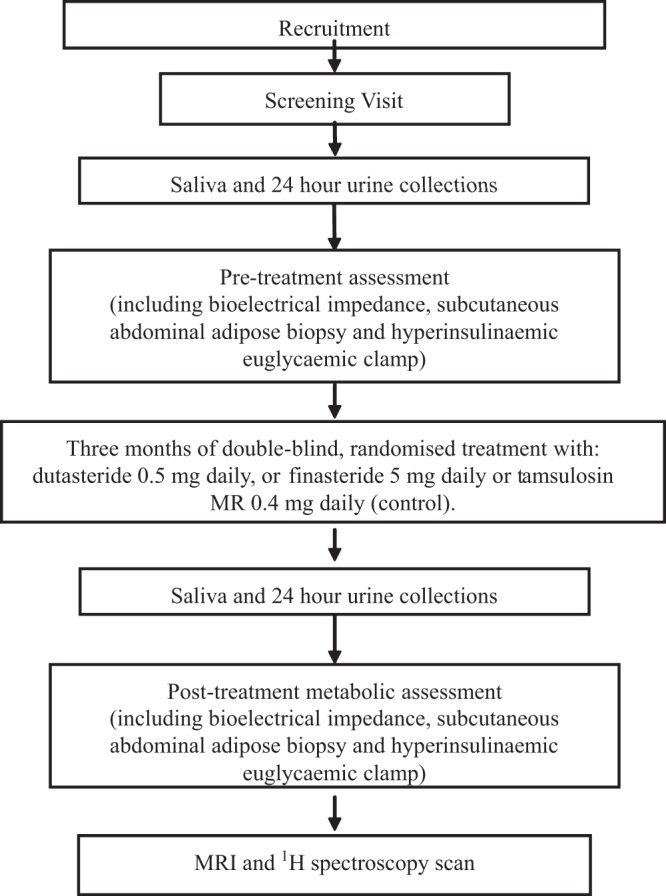
Summary of study protocol.

This was a double-blind, randomized controlled study. Approval from the Lothian Research Ethics Committee and informed written consent were obtained. Participants were studied before and after 3 months of dutasteride (0.5 mg daily; Glaxo Smith Kline Pharmaceuticals), finasteride (5 mg daily; Gedeon Richter), or tamsulosin modified release (MR) (0.4 mg daily; Synthon Hispania) as a control group with doses as used in treatment of BPH (Figure 1). Fixed-size block randomization (n = 18 per block), without stratification or minimization, was performed by Tayside Pharmaceuticals.

### Participants

Participants (age 20–85 years) were recruited from secondary-care urology clinics, primary-care practices, and by advertising. Initial inclusion criteria were men with BPH aged 50 to 80 years, later expanded to all men ≥20 years old to improve recruitment. Exclusion criteria were 5αR inhibitor or glucocorticoid use in previous 3 months; diabetes mellitus or impaired glucose tolerance; significant hepatic, renal, or thyroid disease; hypogonadism; warfarin therapy; body mass index (BMI) ≥40 kg/m^2^; or any suspicion of urological malignancy.

### Outcomes

The primary outcome was insulin sensitivity assessed as glucose disposal during a hyperinsulinemic-euglycemic clamp (11). Secondary endpoints included fasting glucose/insulin relationships, effects of insulin on glucose production and lipolysis, body fat distribution, and gene transcript abundance in sc adipose tissue biopsies. Steroids were measured in blood, urine, and saliva to aid with mechanistic interpretation.

### Clinical methods

Participants collected a 24-hour urine sample and 5 saliva samples (waking, 30 minutes after waking, noon, 4:00 pm, and bedtime) using Salivette collection tubes (Sarstedt) and then attended the Clinical Research Facility at 7:30 am after an overnight fast. Height, weight, blood pressure (BP), pulse rate, and hip and waist circumference were measured using standard techniques. Body fat was measured by bioimpedance using an OMRON BF306 body fat monitor (OMRON Healthcare Ltd). Blood was taken for measurements including glucose, insulin, C-peptide, sex steroids, cortisol, corticosteroid binding globulin (CBG), and adipokines. Biopsies of peri-umbilical sc abdominal adipose were taken with a 14-gauge needle under local anesthesia, with samples snap-frozen on dry ice.

A 3-phase, 2-step hyperinsulinemic-euglycemic clamp was conducted with infusion rates of tracers adjusted for body weight and those for insulin by body surface area as indicated below. From 0 to 90 minutes, only stable isotope tracers (Cambridge Isotope Laboratories, Inc) were infused: 6,6-[^2^H]_2_-glucose (d2-glucose; 17 μmol/kg for 1 minute, then 0.22 μmol/kg/min) and 1,1,2,3,3-[^2^H]_5_-glycerol (d5-glycerol; 1.6 μmol/kg for 1 minute, then 0.11 μmol/kg/min). Tracer infusions were continued, and from 90 to 180 minutes, low-dose insulin was infused (Actrapid; Novo Nordisk; 10 mU/m^2^/min) to measure inhibition of lipolysis and endogenous glucose production. From 180 to 270 minutes, high-dose insulin was infused (40 mU/m^2^/min) with tracers to assess peripheral glucose uptake. During insulin infusion, 20% dextrose (Baxter) infusion was adjusted to maintain euglycemia (4.5 mM–5.5 mM), measured from arterialized samples by glucometer (Accu-Check Advantage; Roche). One sample was taken at baseline, and 4 steady-state samples were taken over 20 minutes at the end of each phase from a hand vein arterialized by external heating of a retrograde cannula (12).

Participants with BPH were unblinded individually on completion of their participation to allow decisions regarding ongoing care. Healthy participants were unblinded at either the interim or final analysis. Adherence was deemed adequate when drug was detected in serum.

### Magnetic resonance imaging and proton magnetic resonance spectroscopy

Magnetic resonance imaging (MRI) and proton magnetic resonance spectroscopy (MRS) measurements of adipose distribution and liver fat, respectively, were undertaken only at the end of the study and not at baseline. Participants without contraindications underwent MRI on a GE Signa Horizon 1.5-T HDxt scanner (General Electric) equipped with a self-shielding gradient set (33 mT m-1 maximum gradient strength) and a manufacturer-supplied torso array coil. Intra-abdominal visceral and sc fat volumes (10-mm slice at L4/5, using iterative decomposition of water and fat with echo asymmetry and least-squares estimation sequence) were quantified using SliceOmatic version 4.3 (TomoVision) software, assuming adipose density of 0.92 g/mL. Single-voxel proton MRS was performed for assessment of hepatic fat, using a point-resolved spectroscopy sequence, with and without water suppression. The voxel (10 mm^3^) was positioned within the liver, avoiding the edge of the liver and major vessels. Spectra were acquired during free breathing, with an echo time of 40 milliseconds and relaxation time of 5000 milliseconds. Postprocessing and quantification of MRS data was performed in jMRUI (13) using a nonlinear least-squares algorithm (Advanced Method for Accurate, Robust and Efficient Spectral fitting, AMARES) (14) with Gaussian line shapes to model each spectral peak of interest (eg, water at 4.7 ppm, methylene fat at 1.3 ppm).

### Laboratory methods

A full blood count was measured on an XE-5000 automated flow cytometer (Sysmex UK); hemoglobin A1c by reverse-phase HPLC (HA8160 analyzer; Menarini); glucose, C-peptide, renal, liver, and thyroid function tests, and lipids by autoanalyzer (Architect c16000 analyzer; Abbott Diagnostics Ltd); serum SHBG by automated chemiluminescent assay (Immulite 2000 system; Siemens); plasma insulin by ultrasensitive ELISA (DRG); salivary cortisol by high-sensitivity ELISA (Salimetrics); plasma cortisol by ^125^I RIA (MP Biomedicals); plasma CBG by (^125^I RIA; DIAsource ImmunoAssays SA); plasma nonesterified fatty acids (NEFAs) by a coupled enzyme reaction assay (Zen-Bio, Inc); plasma leptin, monocyte chemoattractant protein 1, IL-8, adiponectin, and resistin by Milliplex immunoassay (Merck Millipore); and plasma estradiol by chemiluminescent microparticle immunoassay (Abbott Diagnostics) using an Architect c16000 analyzer. Tamsulosin was quantified from serum by liquid chromatography tandem mass spectrometry (LC-MS/MS) (15). Urinary steroids were extracted (16) and analyzed (17) as described previously, with the inclusion of the following transitions (collision energy) for androgens (androsterone, etiocholanolone m/z 360→270, 5α-androstane-3α,17α-diol (internal standard) m/z 331→241 [15 V]). mRNA abundance in sc adipose tissue was determined by real-time quantitative PCR (18), as detailed in Supplemental Table 1, and presented as abundance of gene of interest normalized to the mean of a panel of reference genes (*PPIA*, *TBP*, and *GAPDH*), the abundance of which did not differ between groups.

### Expression of 5αR1 and -2 mRNA in human metabolic tissues

Expression of 5αR1 and 2 mRNA was assessed in human tissues (sc adipose and liver collected with local ethical approval) and in commercially available skeletal muscle cDNA (Primer Design). Total mRNA was extracted using the QIAGEN RNeasy system, and 500 ng was reverse transcribed using the Applied Biosystems high-capacity reverse transcription kit with random primers. cDNA (10 ng) was subjected to PCR with primers specific for 5αR1 or 5αR2 (Supplemental Table 1) using the QIAGEN HotStarTaq Plus system, and products were separated by electrophoresis on a 1.2% agarose gel in 0.5× TBE buffer (Tris base, boric acid, EDTA).

### Supplemental laboratory methods

Serum testosterone, DHT, finasteride, and dutasteride were quantified by LC-MS/MS (Supplemental Table 2), and plasma (during the euglycemic clamp) glucose, d2-glucose, glycerol, and d5-glycerol were quantified by gas chromatography/mass spectrometry.

### Tracer kinetic calculations

Tracer kinetics during the hyperinsulinemic-euglycemic clamp were calculated from average values in steady state: M value = glucose infusion rate at steady state; rate of disposal (Rd) of glucose = d2-glucose infusion rate/tracer-to-tracee ratio; endogenous glucose production (EGP) = Rd glucose − glucose infusion rate; and rate of appearance (Ra) of glycerol = d5-glycerol infusion rate/tracer-to-tracee ratio.

Corrections were applied to adjust the peaks areas of d2-glucose for naturally occurring mass+2 glucose. Infusion rates were calculated specifically for mass+0 glucose and also d2-glucose.

### Sample size and statistical analysis

A power calculation using previously published data (19) predicted 90% power to detect a 15% difference in glucose disposal rates to *P* < .05 with a sample size of 26 per group. A target group size of 33 allowed for a >20% dropout rate. A single planned interim analysis was conducted when at least half the planned participants had completed the study (n = 38). M values (mean steady-state glucose infusion rate) during hyperinsulinemia were analyzed, with *P* < .016 (*P* < .05/3) deemed sufficient for stopping the study. Interim data demonstrated a decrease in insulin sensitivity with dutasteride compared with finasteride (*P* = .002) and tamsulosin (*P* = .003). Therefore, recruitment was stopped and measurements for current participants completed in a final analysis. Analyses were specified a priori; therefore, no statistical adjustment was made for repeated analysis of M values. Results are presented from the final analysis.

Statistical analysis was performed using SPSS for Windows, version 19 (IBM). Areas under the curve were calculated with Kinetica version 5.0 (Thermo Fisher Scientific). Data are presented as mean (SEM) unless stated otherwise. Analysis of covariance was not suitable because the primary and many secondary endpoints did not meet necessary statistical assumptions. ANOVA was therefore conducted on absolute change in each variable from baseline, with least significant difference (LSD) post hoc testing if ANOVA was significant (*P* < .05). If nonnormally distributed data could not be normalized by transformation, then Kruskal-Wallis testing was used. MRI scans were after treatment only, with absolute data rather than change from baseline analyzed by ANOVA as above. Values below the detection limit were considered to be one-third of the limit of detection for each assay. Missing values are indicated and were not imputed. Correlations with age were tested by Pearson correlation.

## Results

### Participant recruitment, characteristics, and withdrawals

Recruitment is summarized in Supplemental Figure 1. Fifty-one men consented, 47 completed the study, and 46 deemed adherent were included in the final analysis. Reasons for withdrawal were subclinical hypothyroidism (n = 1), side effects from study medication (urinary retention and impotence, n = 1), and unrelated illness before commencing study medication (n = 2). One BPH patient developed intolerable urinary symptoms upon cessation of his usual tamsulosin; he was able to complete the study with the addition of rescue tamsulosin to his study medication. Study medications were detected in serum for all but 1 participant (from the dutasteride group) who was deemed nonadherent and excluded from the final analysis. Serum concentrations in others were from 3.0 to 28.5 ng/mL (dutasteride), 2.0 to 64.0 ng/mL (finasteride), and 1.7 to 15.2 ng/mL (tamsulosin).

Characteristics of participants at baseline are summarized in Table 1. Eleven participants were BPH patients (7 were being treated with α-blockers when recruited). Twelve participants were receiving concomitant regular medications, including simvastatin, aspirin, bendroflumethiazide, losartan, lansoprazole, and levothyroxine.

**Table 1.** Characteristics of Study Participants at Baseline^a^

	Dutasteride	Finasteride	Tamsulosin
n	16	16	14
Age, y	35.3 (14.6)	40.3 (19.2)	49.4 (18.4)
Range	20–64	21–85	21–73
BPH patients, n	2	4	5
BMI, kg/m^2^	25.3 (4.4)	26.8 (3.8)	25.5 (2.8)
WHR	0.90 (0.08)	0.89 (0.07)	0.93 (0.06)
Systolic BP, mm Hg	131 (11)	136 (15)	139 (18)
Diastolic BP, mm Hg	78 (10)	78 (11)	81 (9)
Body fat, %	19.8 (8.5) (n = 15)	22.1 (6.6)	24.7 (6.0)
Fasting plasma/serum			
Glucose, mM	5.0 (0.5)	5.0 (0.5)	5.1 (0.4)
Insulin, pM	59 (24)	54 (18)	63 (33)
C-peptide, pM	539 (157) (n = 15)	539 (173)	613 (296)
HOMA-IR	1.89 (0.79)	1.73 (0.63)	2.19 (1.14)
Total cholesterol, mM	4.4 (0.6)	4.9 (1.0)	5.2 (1.0)
Triglycerides, mM	1.2 (0.6)	1.2 (0.7)	1.4 (0.7)

a Data are mean (SD).

### Effects of 5αR inhibition on insulin sensitivity

As shown in Tables 2 and 3, insulin infusion had the predicted effects to suppress EGP and lipolysis (glycerol turnover and NEFA levels) and to stimulate glucose uptake.

**Table 2.** Effects of Drug Interventions on Indices of Insulin Sensitivity for Glucose Metabolism^a^

	Dutasteride (n = 16)			Finasteride (n = 16)			Tamsulosin (n = 14)			*P*, ANOVA
	Before	After	Change	Before	After	Change	Before	After	Change	
Fasting before infusion										
Glucose, mM	5.0 (0.1)	5.1 (0.1)	0.1 (0.1)	5.0 (0.1)	4.9 (0.1)	−0.1 (0.1)	5.1 (0.1)	5.1 (0.1)	−0.1 (0.1)	.34
Insulin, pM	59 (6)	69 (8)	10 (4)	54 (4)	58 (5)	4 (3)	63 (9)	59 (9)	−4 (5)	.07
C-peptide, pM	539 (41)	615 (44)	76 (26)^b,d^	539 (43)	526 (41)	−13 (29)	613 (79)	588 (72)	−24 (34)	.04
HOMA-IR	1.89 (0.20)	2.28 (0.27)	0.39 (0.15)^c^	1.73 (0.16)	1.83 (0.16)	0.10 (0.10)	2.09 (0.31)	1.95 (0.32)	−0.14 (0.16)	.03
During tracer infusion without insulin infusion										
Glucose, mM	5.4 (0.1)	5.3 (0.1)	0.0 (0.1)	5.4 (0.1)	5.3 (0.1)	−0.1 (0.1)	5.6 (0.1)	5.5 (0.1)	−0.1 (0.1)	.89
Insulin, pM	32 (3)	37 (5)	6 (3)^c^	36 (3)	35 (3)	−1 (2)	37 (5)	31 (3)	−6 (3)	.03
EGP, μmol/kg FFM/min	9.03 (0.51)	9.10 (0.55)	0.07 (0.20)	10.23 (0.44)	10.02 (0.46)	−0.21 (0.28)	9.83 (0.58)	10.21 (0.47)	0.38 (0.23)	.24
During low-dose insulin infusion										
Glucose, mM	5.2 (0.1)	5.1 (0.0)	−0.1 (0.1)	5.1 (0.1)	5.0 (0.1)	0.0 (0.1)	5.2 (0.1)	5.1 (0.1)	−0.2 (0.1)	.96
Insulin, pM	86 (9)	91 (10)	5 (11)	84 (6)	83 (6)	−1 (7)	82 (7)	82 (6)	−1 (5)	.84
M value, μmol/kg FFM/min	7.84 (1.72)	8.04 (1.57)	−0.02 (2.32)	9.06 (2.29)	9.13 (1.70)	0.08 (2.01)	6.72 (2.20)	7.12 (1.80)	0.40 (1.16)	.99
EGP, μmol/kg FFM/min	5.10 (0.99)	5.54 (0.77)	0.44 (0.71)	5.80 (0.77)	5.54 (0.86)	−0.25 (0.65)	6.67 (0.64)	6.56 (0.79)	−0.11 (0.52)	.72
During high-dose insulin infusion										
Glucose, mM	4.9 (0.1)	4.9 (0.0)	−0.1 (0.1)	4.9 (0.1)	4.9 (0.1)	0.0 (0.1)	5.0 (0.1)	4.8 (0.1)	−0.2 (0.1)	.53
Insulin, pM	307 (18)	326 (14)	20 (16)	295 (13)	303 (19)	8 (16)	259 (15)	292 (15)	33 (12)	.51
M value, μmol/kg FFM/min	45.2 (4.0)	39.0 (4.8)	−6.2 (3.4)^c,e^	40.0 (4.1)	47.8 (5.1)	7.8 (3.2)	30.7 (4.2)	38.3 (4.7)	7.6 (2.2)	.002
Rd glucose, μmol/kg FFM/min	41.9 (3.54)	36.1 (4.41)	−5.7 (3.2)^c,e^	37.0 (3.77)	44.2 (4.74)	7.2 (3.0)	28.4 (3.88)	35.4 (4.38)	7.0 (2.0)	.002

Abbreviation: FFM, fat-free mass.

a Data are mean (SEM) of the values from each study day obtained at baseline after overnight fast and as an average of 4 measurements in 15-minute steady-state periods after low-dose or high-dose insulin infusions (n = 11–12 per group). Steady state was confirmed, with the relative SD of the tracer-to-tracee ratios of d2-glucose to glucose between 0.4% and 3.8%. M value is the glucose infusion rate at steady state. ANOVA was conducted on absolute change in each variable from baseline, with LSD post hoc testing if ANOVA was significant (*P* < .05).

b *P* < .05 vs tamsulosin.

c *P* ≤ .01 vs tamsulosin.

d *P* < .05 vs finasteride.

e *P* ≤ .01 vs finasteride.

**Table 3.** Effects of Drug Interventions on Lipid Profile and Insulin Sensitivity for Lipolysis^a^

	Dutasteride (n = 16)			Finasteride (n = 16)			Tamsulosin (n = 14)			*P*, ANOVA
	Before	After	Change	Before	After	Change	Before	After	Change	
Total cholesterol, mM	4.4 (0.2)	4.3 (0.2)	−0.1 (0.1)	4.9 (0.3)	5.0 (0.3)	+0.1 (0.1)	5.2 (0.3)	4.9 (0.3)	−0.3 (0.1)	.08
HDL-cholesterol, mM	1.3 (0.1)	1.3 (0.1)	−0.0 (0.0)	1.3 (0.1)	1.3 (0.1)	0.0 (0.0)	1.4 (0.2)	1.3 (0.1)	−0.1 (0.1)	.47
LDL-cholesterol, mM	2.6 (0.2)	2.6 (0.2)	+0.0 (0.1)	3.0 (0.3)	3.1 (0.3)	+0.1 (0.1)	3.2 (0.2)	3.1 (0.3)	−0.1 (0.2)	.43
Triglycerides, mM	1.3 (0.1)	1.1 (0.2)	−0.2 (0.1)	1.2 (0.2)	1.2 (0.2)	+0.0 (0.1)	1.4 (0.2)	1.2 (0.2)	−0.1 (0.1)	.38
Fasting before infusion										
Glycerol, μM	48.4 (5.3)	67.9 (18.4)	19.5 (14.5)	50.4 (7.2)	47.7 (7.0)	−2.7 (7.3)	53.6 (9.5)	42.3 (5.4)	−11.3 (5.8)	.10
NEFAs, μM	485.1 (53.2)	523.8 (69.7)	38.7 (73.7)	443.9 (54.8)	493.1 (43.8)	49.2 (51.2)	649.4 (71.4)	580.2 (102.3)	−69.2 (61.6)	.37
During tracer infusion without insulin infusion										
Glycerol, μM	41.2 (5.0)	45.7 (6.3)	+4.5 (5.2)	42.8 (4.7)	38.4 (4.7)	−4.4 (4.2)	42.3 (6.3)	38.2 (4.3)	−4.0 (3.7)	.28
Ra glycerol, μmol/kg FFM/min	2.54 (0.34)	2.78 (0.42)	0.24 (0.21)	2.32 (0.28)	2.27 (0.16)	−0.04 (0.25)	3.14 (0.39)	3.04 (0.39)	−0.09 (0.30)	.60
NEFAs, μM	484.2 (52.5)	520.1 (51.7)	35.9 (49.8)	479.6 (58.4)	497.5 (63.3)	17.9 (51.5)	628.9 (59.8)	577.2 (53.3)	−51.7 (51.2)	.46
During low-dose insulin infusion										
Glycerol, μM	18.0 (2.6)	22.4 (4.0)	4.4 (2.6)	17.4 (3.1)	16.0 (3.0)	−1.5 (3.1)	22.6 (6.7)	16.0 (2.6)	−6.7 (6.1)	.17
Ra glycerol, μmol/kg FFM/min	1.23 (0.15)	1.39 (0.17)	0.16 (0.15)	1.32 (0.18)	1.30 (0.17)	−0.02 (0.10)	1.99 (0.36)	1.60 (0.12)	−0.39 (0.32)	.16
NEFAs, μM	184.9 (27.9)	245.2 (36.6)	60.3 (30.0)^b^	193.4 (32.9)	189.7 (33.6)	−3.7 (14.9)	295.1 (56.2)	214.1 (22.1)	−81.0 (60.4)	.04
During high-dose insulin infusion										
Glycerol, μM	14.7 (2.67)	13.1 (2.87)	−1.8 (1.3)	10.6 (2.39)	8.9 (2.14)	−1.7 (2.0)	15.0 (5.56)	6.9 (1.98)	−8.1 (4.5)	.21
NEFAs, μM	36.3 (4.0)	39.0 (5.9)	2.3 (5.9)	37.1 (4.5)	37.8 (4.9)	0.7 (3.7)	53.3 (9.8)	32.2 (2.8)	−21.1 (9.8)	.12

Abbreviations: FFM, fat-free mass; HDL, high-density lipoprotein; LDL, low-density lipoprotein.

a Data are mean (SEM) of the values from each study day obtained at baseline after overnight fast and as an average of 4 measurements in 15-minute steady-state periods after low-dose or high-dose insulin infusions (n = 11–12 per group). Steady state was confirmed, with the relative SD of the tracer to tracee ratios of d5-glycerol to glycerol between 1.2% and 11.7%. ANOVA was conducted on absolute change in each variable from baseline, with LSD post hoc testing if ANOVA was significant (*P* < .05).

b *P* ≤ .01 vs tamsulosin.

**Figure 2.**
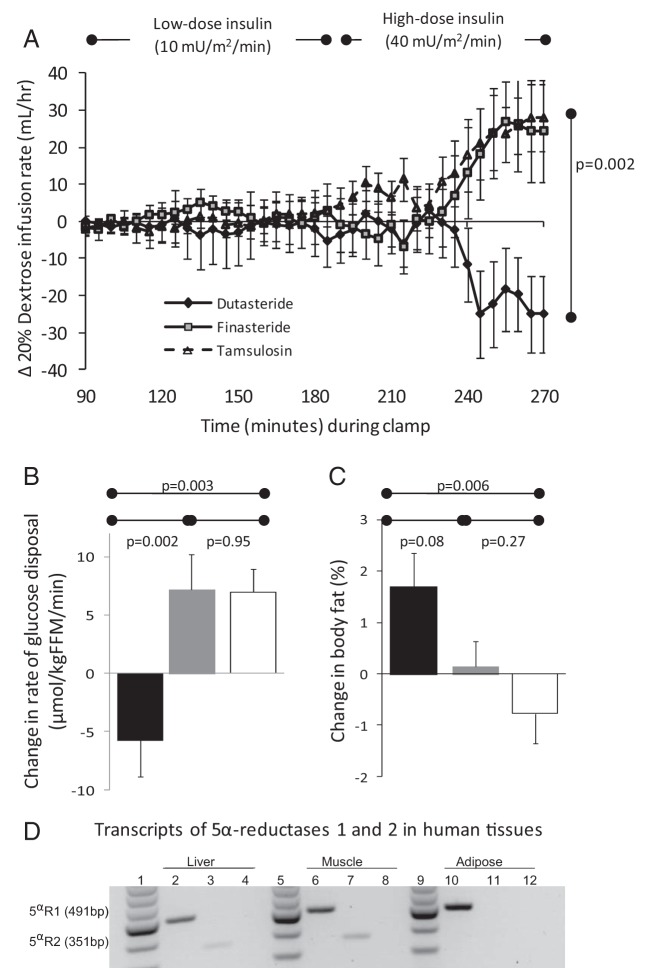
Effects of 5αR inhibition on insulin sensitivity and body fat. A, Change in glucose (20% dextrose) infusion rate (milliliters per hour) required to maintain euglycemia during low-dose (10 mU/m^2^/min) and high-dose (40 mU/m^2^/min) insulin infusion. Data are mean (SEM). B, Change in glucose Rd during high-dose insulin infusion after dutasteride (black), finasteride (gray), or tamsulosin (white) treatment. Data are mean (SEM). C, Change in percent body fat measured by electrical bioimpedance after dutasteride (black), finasteride (gray), or tamsulosin (white) treatment. Data are mean (SEM). D, Transcripts of 5αR1 and -2 in human liver, skeletal muscle, and sc adipose tissue. Lanes 1, 5, and 9, 100-bp ladder; lanes 2, 6, and 10, 5αR1; lanes 3, 7, and 11, 5αR2; lanes 4, 8, and 12, negative control. Abbreviation: FFM, fat-free mass.

Dutasteride, but not finasteride or tamsulosin, markedly decreased the glucose Rd (M value, the primary endpoint) during high-dose insulin infusion (Figure 2, A and B, and Supplemental Figure 2 and Table 2), increased fasting plasma C-peptide and homeostatic model assessment of insulin resistance (HOMA-IR) (Table 2), and increased plasma insulin levels when tracers were infused alone (Table 2). EGP during low-dose insulin was unaffected by study drugs (Table 2). Given the wide age range of participants, we tested whether age influenced the primary endpoint; the change in M value after drug treatment, measured during high-dose insulin infusion, did not correlate with age (dutasteride *r* = −0.28, *P* = .31; finasteride *r* = 0.17, *P* = .53; tamsulosin *r* = −0.13, *P* = .66).

Dutasteride, but not finasteride or tamsulosin, impaired suppression of plasma NEFA levels during low-dose insulin infusion only, although glycerol turnover was unaffected by drug treatment (Table 3).

### Effect of 5αR inhibition on body composition and adipose tissue

There were no effects of drug treatment on BP, heart rate, body weight, BMI, or waist-to-hip ratio (WHR) (Table 4). There was, however, an increase in body fat (measured in kg or %) with dutasteride, but not finasteride, compared with tamsulosin (Figure 2C, Table 4). The increase in body fat with dutasteride was not accompanied by measurable differences in visceral or subcutaneous abdominal adipose volume on MRI (Table 4). Liver fat fraction (by MRS) was not measured at baseline and was compared only at the end of the study, when it was not different between treatment groups, either with (*P* = .22) or without adjustment for potential confounders (body weight, BMI, body fat, and WHR): median (interquartile ranges), dutasteride 9.4% (3.6, 23.6; n = 13), finasteride 4.7% (1.3, 43.2; n = 15), and tamsulosin 3.4% (1.8, 9.2; n = 9).

**Table 4.** Effects of Drug Interventions on Body Fat and BP^a^

	Dutasteride (n = 16; body fat n = 15; MRI n = 13)			Finasteride (n = 16)			Tamsulosin (n = 14; MRI n = 11)			*P*, ANOVA
	Before	After	Change	Before	After	Change	Before	After	Change	
Weight, kg	77.4 (3.2)	78.3 (3.0)	+1.0 (0.6)	83.8 (3.5)	83.2 (3.4)	−0.6 (0.5)	80.5 (2.8)	80.5 (3.0)	0.0 (0.7)	.17
BMI, kg/m^2^	25.3 (1.1)	25.6 (1.0)	+0.3 (0.2)	26.8 (1.0)	26.6 (0.9)	−0.2 (0.2)	25.5 (0.7)	25.6 (0.9)	+0.1 (0.2)	.14
WHR	0.90 (0.02)	0.90 (0.02)	0.00 (0.01)	0.89 (0.02)	0.90 (0.01)	+0.00 (0.01)	0.93 (0.02)	0.93 (0.01)	0.00 (0.01)	.96
Systolic BP, mm Hg	131 (3)	135 (4)	+4 (4)	136 (4)	140 (2)	+4 (3)	139 (5)	138 (4)	−1 (4)	.56
Diastolic BP, mm Hg	78 (3)	82 (2)	+4 (2)	78 (3)	80 (2)	+2 (2)	81 (2)	82 (2)	+1 (2)	.60
Body fat, kg	16.5 (2.1)	17.8 (2.1)	+1.2 (0.4)^b^	18.9 (1.7)	18.7 (1.6)	−0.2 (0.5)	20.1 (1.6)	19.6 (1.8)	−0.5 (0.6)	.048
Body fat, %	19.8 (2.1)	21.5 (2.0)	+1.6 (0.6)^c^	22.1 (1.7)	22.3 (1.7)	+0.2 (0.5)	24.7 (1.6)	24.0 (1.9)	−0.8 (0.6)	.02
Visceral fat, kg		0.09 (0.01)			0.07 (0.01)			0.10 (0.02)		.16
sc fat, kg		0.22 (0.03)			0.21 (0.02)			0.20 (0.02)		.85

a Data are mean (SEM). Visceral and sc fat was measured in a cross-section at L4/5. ANOVA was conducted on absolute change in each variable from baseline, with LSD post hoc testing if ANOVA was significant (*P* < .05).

b *P* < .05 vs tamsulosin.

c *P* ≤ .01 vs tamsulosin.

There were no differences in serum lipid profile (Table 3) and no drug-induced changes in serum adipokines (leptin, adiponectin, or resistin) or cytokines (monocyte chemoattractant protein 1 or IL-8) (Supplemental Table 3). In sc adipose, androgen receptor mRNA decreased from baseline in both dutasteride- and finasteride-treated groups compared with tamsulosin (Supplemental Table 4), but no other transcripts tested were altered.

### Effects of 5αR inhibitors on steroid profile

Both dutasteride and finasteride, but not tamsulosin, decreased serum DHT and decreased urinary excretion of the A-ring-reduced metabolites of both androgens and glucocorticoids to a similar extent (Table 5). Steroid binding globulins, and cortisol in plasma (Table 5) and saliva (Supplemental Figure 3) did not differ between groups. There was a trend for 5αR inhibitors to increase estradiol levels in blood.

**Table 5.** Effects of Drug Interventions on Steroids in Plasma and Urine^a^

	Dutasteride (n = 16)			Finasteride (n = 16)			Tamsulosin (n = 14)			*P*, ANOVA
	Before	After	Change	Before	After	Change	Before	After	Change	
Circulating steroids and binding proteins										
Testosterone, nM	25 (2)	30 (3)	+5 (2)	21 (2)	24 (2)	+3 (1)	21 (2)	23 (3)	+2 (1)	.22
DHT, nM	2.9 (0.4)	1.8 (0.4)	−1.1 (0.2)^b^	2.1 (0.3)	1.0 (0.2)	−1.1 (0.2)^b^	2.0 (0.3)	1.7 (0.3)	−0.3 (0.2)	.02
Cortisol, nM	788 (59)	692 (47)	−96 (40)	769 (51)	689 (43)	−80 (54)	818 (54)	757 (42)	−61 (55)	.88
Estradiol, pM	81.8 (7.9)	126.7 (16.9)	+44.9 (15.8)	69.3 (7.4)	94.0 (8.9)	+24.8 (6.9)	73.4 (9.4)	80.8 (10.9)	+7.4 (7.5)	.07
SHBG, nM	28 (2)	27 (3)	−1 (1)	24 (2)	25 (2)	+1 (1)	31 (3)	33 (4)	+2 (2)	.25
CBG, nM	988 (43)	966 (42)	−22 (36)	936 (32)	905 (53)	−31 (50)	959 (34)	929 (42)	−31 (23)	.75^d^
Albumin, g/L	42 (1)	39 (1)	−3 (1)	43 (1)	40 (1)	−3 (1)	41 (1)	40 (1)	−1 (1)	.26
Urinary steroids										
Androsterone (α), μg/d	1806 (175)	121 (18)	−1684 (163)^b^	2373 (434)	397 (78)	−1975 (397)^b^	2116 (347)	2036 (336)	−79 (268)	<.001
Etiocholanolone (β), μg/d	817 (101)	2461 (338)	+1643 (283)^b^	805 (151)	1710 (251)	+905 (198)^b^	861 (130)	1029 (176)	+168 (99)	<.001^d^
5α-THF, μg/d	1664 (252)	32 (9)	−1633 (249)^b^	1858 (356)	51 (13)	−1807 (346)^b^	1786 (300)	1773 (308)	−13 (200)	<.001
5β-THF, μg/d	1724 (128)	1718 (130)	−6 (127)	1670 (162)	1683 (127)	+13 (159)	1793 (136)	1742 (165)	−52 (199)	.96
β-THF/α-THF	1.39 (0.20)	96.20 (16.68)	+94.81 (16.75)^b^	1.21 (0.18)	55.69 (8.34)	+54.48 (8.32)^b^	1.30 (0.18)	1.37 (0.24)	+0.07 (0.12)	<.001^d^
F/α-THF	0.10 (0.03)	6.12 (0.92)	+6.01 (0.92)^b^	0.10 (0.01)	4.22 (0.58)	+4.11 (0.58)^b^	0.12 (0.03)	0.13 (0.03)	+0.01 (0.02)	<.001^d^
F/β-THF	0.08 (0.01)	0.08 (0.01)	0.00 (0.01)	0.09 (0.01)	0.08 (0.01)	−0.01 (0.00)	0.09 (0.01)	0.09 (0.01)	0.00 (0.01)	.67
Etiocholanolone/androsterone	0.45 (0.05)	21.82 (2.43)	+21.37 (2.43)^b,c^	0.36 (0.05)	8.26 (3.89)	+7.89 (3.88)^b^	0.43 (0.04)	0.85 (0.39)	+0.43 (0.38)	<.001^d^

Abbreviations: F, cortisol; THF, tetrahydrocortisol.

a Data are mean (SEM). ANOVA was conducted on absolute change in each variable from baseline, with LSD post hoc testing if ANOVA was significant (*P* < .05).

b *P* ≤ .01 vs tamsulosin.

c *P* ≤ .01 vs finasteride.

d Kruskal-Wallis test with pairwise comparisons.

### Expression of 5αR isozymes in human tissues

Transcripts of both 5αR1 and 5αR2 were detected in human liver and skeletal muscle, but only 5αR1 mRNA was detected in sc adipose tissue (Figure 2D).

## Discussion

These data highlight a previously unrecognized role of 5αR1 in modulating metabolic signaling in humans and detail the metabolic sequelae of 5αR inhibition in men. We demonstrate an increase in body fat and decrease in insulin sensitivity induced by the dual 5αR1/5αR2 inhibitor dutasteride, but not by the selective 5αR2 inhibitor finasteride, despite similar effects on circulating and urinary steroids. The metabolic effects of dutasteride are mediated in peripheral tissues, most likely including adipose tissue where 5αR1 but not 5αR2 is expressed. We therefore attribute these effects principally to inhibition of 5αR1 and consequent altered tissue steroid concentrations; this is supported by a recent publication demonstrating an adverse metabolic phenotype in 5αR1-deficient mice (9).

Although 5αR inhibitors have been used extensively clinically, previous studies of metabolism with 5αR inhibition (10, 20, 21) have neither been randomized nor adequately controlled; nor have they incorporated sensitive measures of insulin sensitivity. A crossover study is not feasible due to the long half-life of dutasteride (5 weeks) (22). We therefore designed a parallel-group randomized study to conduct detailed metabolic investigations and included a control group treated with tamsulosin, which is not known to have metabolic effects but allowed for inclusion of patients with symptomatic BPH.

The principal site of 5αR1 expression outside of the skin is the liver (2). Mice with life-long deficiency in 5αR1 exhibit liver fat accumulation after metabolic challenge (9), and we anticipated that effects of dutasteride on whole-body insulin sensitivity may be accompanied by liver fat accumulation and impaired suppression of EGP by insulin in the liver, with corresponding changes in serum lipid profile. However, our data in healthy men after dutasteride treatment for 3 months suggest preservation of hepatic insulin sensitivity after 5αR1 inhibition. Although in rodents 5αR1 is the predominant isozyme in liver, in humans, both 5αR1 and 5αR2 are expressed in liver (2), and their relative roles have not previously been described. We found that finasteride and dutasteride have similar effects on excretion of urinary 5α-reduced androgens and glucocorticoids, which reflect the intrahepatic steroid milieu as they are excreted as conjugates formed in the liver. The only difference we observed in steroid profiles between finasteride and dutasteride was a modestly higher etiocholanolone/androsterone ratio with dutasteride. This suggests that 5αR1 makes only a limited contribution, over and above that of 5αR2, to liver steroid metabolism in humans.

Whereas hepatic insulin sensitivity was preserved, dutasteride strikingly decreased glucose disposal during high-dose insulin infusion, consistent with impaired insulin sensitivity in peripheral organs, including skeletal muscle and/or adipose tissue. This contrasted with an improvement in peripheral insulin sensitivity after 3 months treatment in the finasteride and tamsulosin groups, potentially explained by the Hawthorne effect of improved health during participation in clinical studies (23). We confirmed previous reports that 5αR1 is expressed in human skeletal muscle (4), but we did not assess skeletal muscle metabolism further here.

Dutasteride increased body fat and reduced insulin-mediated suppression of NEFAs, consistent with impaired insulin sensitivity in adipose tissue. We could not attribute the increase in body fat to a specific change in sc, visceral, or hepatic adiposity, but this may reflect lack of statistical power for these secondary endpoints, particularly because MRI and proton MRS were not performed in every participant or at baseline. We did not demonstrate altered whole-body lipolysis by d5-glycerol turnover, but this may reflect biological or analytical variability. Alternatively, there may be an effect on fatty acid esterification, but this could not be demonstrated without the use of a palmitate tracer. We showed, using PCR, that 5αR1 but not 5αR2 is expressed in human adipose tissue. No alterations were found in intra-adipose transcript abundance or circulating adipokines that are likely to account for impaired insulin sensitivity; the observed reduction in androgen receptor mRNA might be metabolically adverse (24) but was observed with both finasteride and dutasteride so is most likely a response to altered circulating androgen levels. However, only sc adipose tissue was biopsied, whereas steroid signaling may exert greater effects in visceral adipose tissue. Taken together, these observations are consistent with metabolic effects of dutasteride being mediated by inhibition of 5αR1 in adipose tissue but do not exclude either a contribution from other tissues including skeletal muscle or a contribution from more potent inhibition of 5αR2 by dutasteride than finasteride.

A third isozyme of 5αR has been described and is expressed in relevant tissues (25, 26). Its role in steroid metabolism is as yet not clearly defined, and furthermore, effects of 5αR inhibitors on this isozyme are uncertain (27), and any relevance to our findings is unclear.

Previous studies have shown more potent effects of dutasteride than finasteride to lower circulating DHT levels (10, 28). Here, despite a higher etiocholanolone/androsterone ratio in urine, suggesting somewhat more potent overall 5αR inhibition by dutasteride, we did not find any differences in circulating DHT. This may reflect our use of a highly specific LC-MS/MS assay, although we might have obtained different results after longer-term treatment given the long half-life and very slow time to steady state for dutasteride (29). Most importantly, this indicates that differences in effects of dutasteride and finasteride on insulin sensitivity are not mediated by differences in circulating DHT. More studies are now justified to assess tissue steroid hormone concentrations and identify the downstream signaling pathways affected, particularly in adipose tissue and skeletal muscle. Such studies could test the hypotheses that either decreased androgen action and/or increased glucocorticoid action mediates these effects.

These results highlight a novel role for 5αR1 on modulating human metabolism; however, their clinical relevance is uncertain. The decrease in insulin sensitivity after 3 months of dutasteride (∼14%) is of similar magnitude to the beneficial effects of antidiabetic agents such as metformin (30). Impaired insulin sensitivity measured by euglycemic clamps predicts future risk of type 2 diabetes mellitus (31). Our study sample consisted mostly of healthy men who are younger than those affected by BPH with declining β-cell function (32) and increased body fat (33). Importantly, age did not confound the effect of dutasteride on insulin sensitivity in the study. Nonetheless, older men with already impaired insulin sensitivity might be more susceptible to the metabolic consequences of 5αR inhibition; the effect of disruption of 5αR1 in murine models is revealed with a high-fat diet (9). The association of BPH with the metabolic syndrome (34, 35), and the likelihood of long-term exposure to 5αR inhibitors once treatment is initiated, suggests that further studies should now be conducted to establish whether inhibition of 5αR1 has clinically important effects on adiposity and metabolism in men with BPH.
